# Study of changes in the aging process, microstructure, and mechanical properties of AA2024–AA1050 nanocomposites created by the accumulative roll bonding process, with the addition of 0.005 vol.% of alumina nanoparticles

**DOI:** 10.1186/s11671-023-03917-2

**Published:** 2024-01-02

**Authors:** Hamed Roghani, Ehsan Borhani, Ehsan Ahmadi, Hamid Reza Jafarian

**Affiliations:** 1 Nanomaterials Department, Faculty of New Sciences and Technologies, Semnan University, Semnan, Iran; 2 School of Metallurgy and Materials Engineering, Iran University of Science and Technology (IUST), Tehran, Iran

**Keywords:** Accumulative roll bonding (ARB), Aluminum, Alumina, Nanocomposites, Aging process, Nanoparticles

## Abstract

We created AA2024–AA1050 and AA2024–AA1050/0.005 vol.% Al_2_O_3_ nanocomposites by six accumulative roll bonding (ARB) process cycles. We used AA2024 and AA1050 sheets with a thickness of 0.7 mm and plate-shaped alumina nanoparticles to create a composite. The two AA1050 and one AA2024 sheets (among the two AA1050 sheets) were ARB-ed up to six cycles with and without adding alumina nanoparticles. Also, a sample of the AA1050 without composite making was ARB-ed up to six cycles. We aged some composites after the ARB process in the furnace at 110, 150, and 190 °C. This project performed SEM, TEM, and EDS-MAP analyses, tensile strength, microhardness, and Pin-on-Disc tests to study the ARB-ed sheets. The results of the tensile tests showed that the tensile strength of AA2024–AA1050 created by the six cycles ARB process was two times more than primary AA1050. Also, the wear resistance of this composite was 74% more than six cycles ARB-ed the AA1050. Using 0.005 vol.% alumina nanoparticles in AA2024–AA1050 composite improved its wear resistance by 30%. In the following, the aging process caused an improvement in tensile strength and total elongation of AA2024–AA1050/Al_2_O_3_ nanocomposites.

## Introduction

Metal aluminum is relatively lightweight (2.7 g/cm^3^), with good ductility and high strength-to-volume fraction. However, pure aluminum's strength is less than other metals, such as steel. Therefore, scientists tried to increase the strength of aluminum by different methods. These methods include solid solution, composite making, aging process, and work hardening. Metallurgists divide aluminum sheets into eight series [1–3].

The 1000 series is an almost pure aluminum alloy. This series cannot be heat-treated. Only fine graining and applying mechanical work can increase the strength of this series of alloys. Series of 1000 aluminum alloy has general applications such as building facades and structural applications. This series of alloys has feeble wear resistance and low strength that limits their application [3–5]. However, composite making with stronger and harder materials can increase these low-cost alloys' wear resistance and tensile strength.

The 2000 series has copper and magnesium alloy elements. This series can heat treated and create Al_2_Cu and Al_2_CuMg precipitates. These precipitates increase the hardness and tensile strength of the aluminum alloys. Series of 2000 aluminum alloys has particular applications in the aerospace and automotive industries [6–9]. These alloys' tensile strength and wear resistance are much higher than the 1000 series. Nevertheless, their price is higher than the 1000 series.

Accumulative roll bonding (ARB) is an efficient process to modify the grains′ structure and increase the tensile strength of metal sheets. In this method, successive rolling with reducing the thickness by about 50% creates an ultrafine-grained (UFG) structure. In the ARB method, work hardening and increase of grain boundaries cause to enhance the tensile strength. According to the Hall–Petch equation, reducing grain size to less than 1 µm (creating an ultrafine-grained structure) will significantly increase grain boundary and, thus, tensile strength [10–13]. To create the UFG microstructure, the von-Mises equivalent strain should be 4 [14, 15]. A 50% thickness decrease in rolling cause strain equivalent to 0.8 von-Mises. Due to a 50% thickness decrease in each ARB cycle, alloys need 5 ARB cycles to achieve the UFG structure. Of course, things like a solid solution, precipitation, and heat treatment between ARB cycles can cause the UFG structure to be created at different strains [11, 15].

The ARB method can create a lamellar composite among dissimilar metal sheets and metal-based composites with strengthening particles. Such of these composites include AA2014/AA6063 [16] Al-Nb/Ti/Ni [17], Al- (TIB_2_ + TiC) P/6063 [18], AA1050/CuO [19], Al/SiC_P_ [20] and Al/Al_2_O_3_ [21].

One can create a layered composite by placing metal layers on each other. The rolling causes the harder layer to crush in layered composites due to the slow strain. However, the soft layers remain continuous due to the high strain rate [22–24]. Adding reinforcement particles in the early cycles of ARB can create a metal composite and reinforcing particles. Increasing the length two-fold in each cycle and putting the layers on each other in the next step will improve the distribution of reinforcement particles [11, 25–27].

Despite the ARB benefits, this method reduces the ductility of metal sheets. Performing the engineered heat treatment after ARB can significantly improve elongation [28]. Also, ARB reduces wear resistance. The reason for this is the layered structure of the ARB-ed sheet and the increase in the mechanism of delamination during wearing. Creating a composite with ceramic reinforcement particles may improve wear resistance [4, 23].

This study created AA1050 (66%)–AA2024 (34%) nanocomposites using AA1050 and AA2024 sheets and the ARB method. Also, we investigated the effect of adding 0.005 vol.% alumina nanoparticles on nanocomposites' microstructure and mechanical properties. Furthermore, after composite creation, the effect of aging at different temperatures and times on the mechanical properties was investigated. The discussion section introduced the rational connection between the microstructure, the mechanical properties, and the mechanism of wearing resistance improvement in the AA2024–AA1050 nanocomposite with Al_2_O_3_ nanoparticles.

## Experimental

**Fig. 1:**
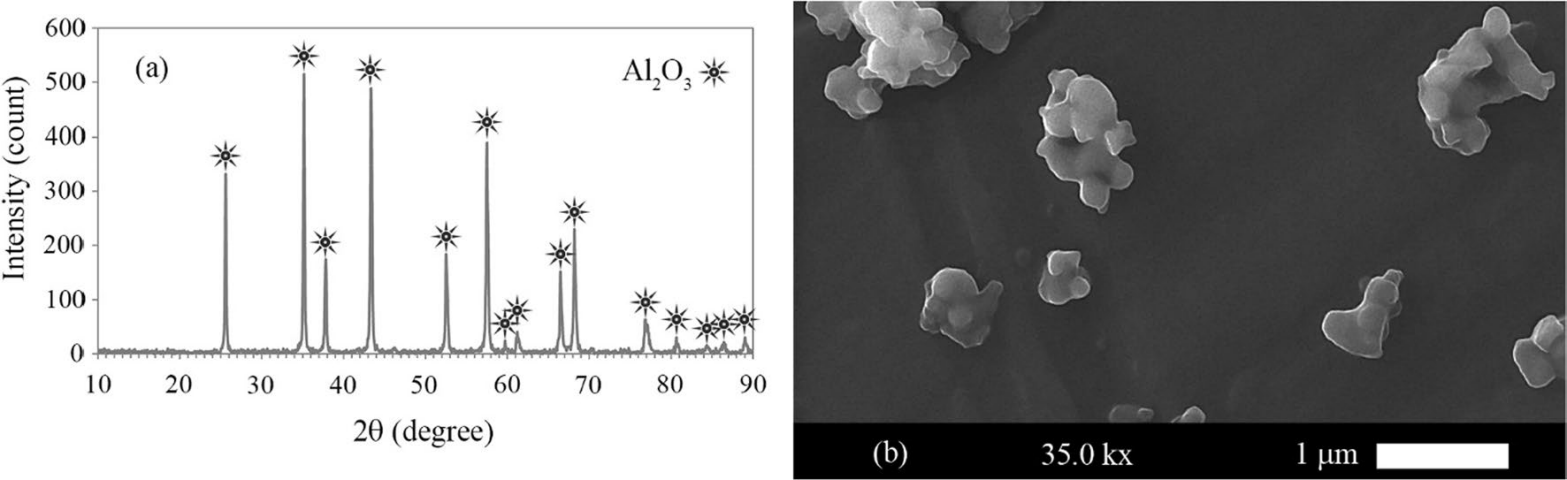
**a** The X-ray diffraction pattern and **b** the scanning electron microscopy image of alumina nanoparticles

This study used the AA1050 (IRALCO—Iranian Aluminium Company) and AA2024 (WMH Group, Germany) sheets with dimensions of 80 × 30 × 0.7 mm. A quantometer measured the elemental composition of the aluminum alloy sheets. Table 1 shows the chemical composition of the two alloys. Also, this study used Al_2_O_3_ nanoparticles (purity of more than 98% and particle size less than 100 nm) as reinforcement. Initially, the particles were agglomerated but separated by successive rolling [23]. Figure 1 shows the XRD and SEM analysis of these nanoparticles.

**Table 1:** The chemical composition of AA1050 and AA2024 sheets

Sheet	Elements	Al	Mg	Cu	Mn	Fe	Si	Ga	V	Zn
2024 Aluminum alloy	wt.%	93.32	1.628	4.25	0.437	0.109	0.0746	0.007	0.007	0.115
1050 Aluminum alloy	wt.%	99.53	0.008	0.016	0.010	0.289	0.062	0.011	0.015	0.027

**Fig. 2:**
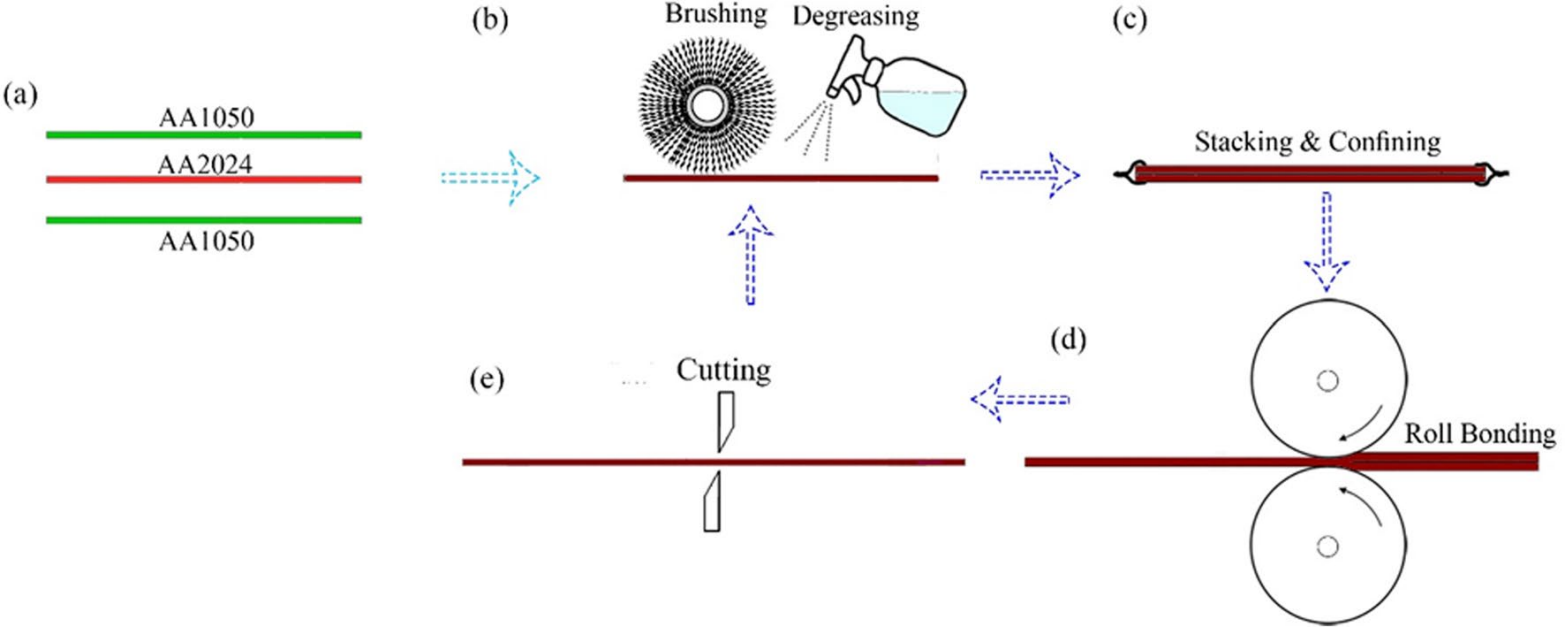
Schematic of the ARB process conducted in this research

Box furnace annealed the 1050 and 2024 aluminum alloy sheets at 370 and 500 °C, respectively. Also, the annealing time for 1050 and 2024 aluminum alloys was six and two hours, respectively. Furthermore, all sheets after the annealing were water quenched. After the annealing, we placed a sheet of AA2024 between the two AA1050 sheets (Fig. 2a). A metal brush in the longitudinal direction scratched the sheet contact surface (Fig. 2b). A copper wire joined the aluminum sheets at the two ends (Fig. 2c), and in the following, rolling was done (Fig. 2d). Rolling was carried out by a device with a diameter of 360 mm, rotation velocity of 12 rounds per minute, and 50% thickness reduction. The first cycle of ARB was performed by doing these processes. After rolling, iron scissors cut the sheet in half. Then, scratching the contact surface, overlapping, connecting by copper, and rolling was done again. We did the rolling in six cycles. In order to investigate the effect of adding alumina, the operator of rolling sprayed acetone suspension and Al_2_O_3_ at vol.% 0.005 before the first cycle of ARB on the two AA2024 surfaces. Figure 2 shows the schematic of the ARB process in this project.

After six ARB cycles, this study performed the aging process at different temperatures and times. Temperature and time of aging are shown in Table 2.

**Table 2:** Temperatures and times of the aging process

Temperature (°C)	Time (h)				
	0.5	1	3	5	10
110	–	–	*	*	*
150	–	*	*	*	–
190	*	*	*	–	–

This study used the Philips XRD device (PW 3710 and Cu Kα) phase investigation. Also, we used the optical microscope of the Leitz Metallux 3 and the field emission scanning electron microscopy (FESEM) Tescan (Mira3-XM) to investigate the microstructure. The Keller solution (2 ml HF acid, 3 ml HCl acid, 5 ml HNO_3_, and 190 ml distilled H_2_O) was used for etching if needed. Furthermore, Matsuzawa, MMT performed microhardness measurement under a force of 0.98 N. The microhardness for each sample was performed on the ND-RD plane at 5 points from the surface to the sample center. The SUT-500K SANAF device measured the tensile strength at a strain rate of 8.3 × 10^−4^ (S − 1). Wire cut machine cut the tensile test samples in the rolling direction according to the JIS Z 2201 standard. The Pin-on-Disc experiment also checked resistance to wear. The details of the Pin-on-Disc are in Table 3.

**Table 3:** Specifications of pin-on-disc test

Characteristic	Quantity
Force	2 N
Radius	0.0065 m
Distance	400 m
Speed	0.12 m/s
Radius per minute	176

## Results

### AA2024–AA1050 nanocomposites microstructure produced by ARB method

**Fig. 3:**
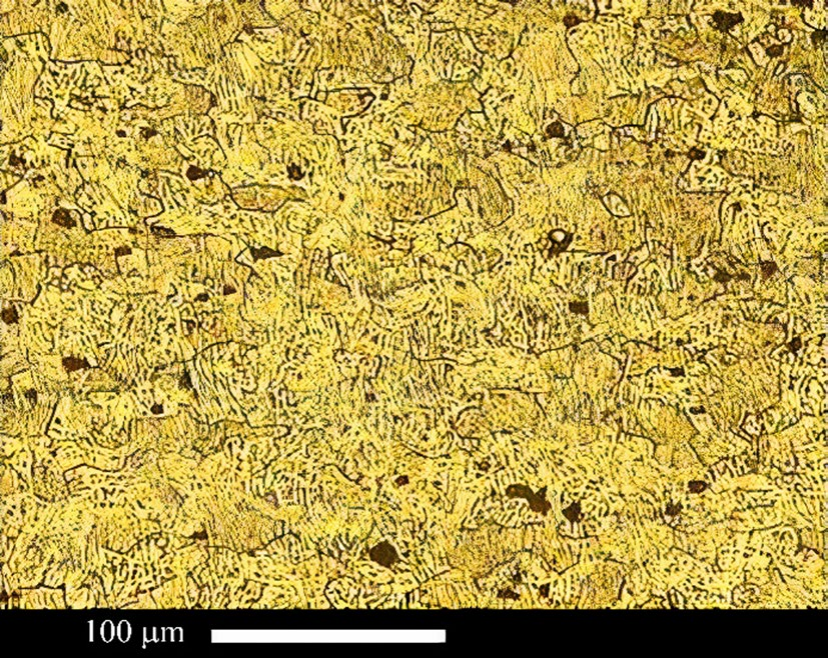
Optical microscopy picture of annealed 2024 aluminum alloy

Figure 3 shows the optical microscope of the AA2024 alloy after annealing. The optical microscope picture shows that Al grains are 2–30 µm wide. Aluminum 1050 alloy was similar to our past work [3, 19]. According to past work, the AA1050 comprises equiaxed micrometer grains with a 5–40 µm diameter.

**Fig. 4:**
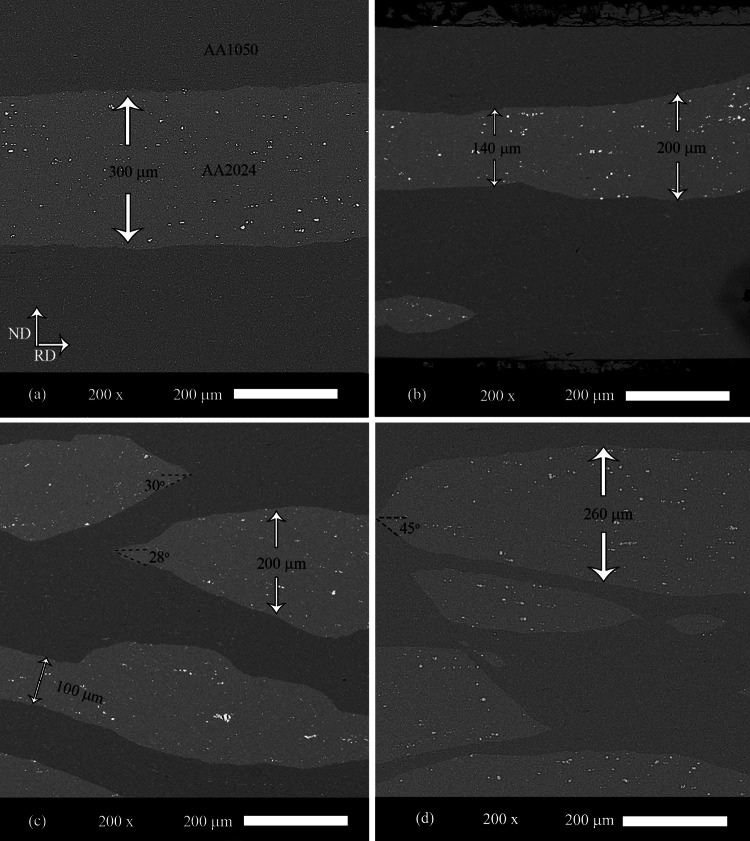
SEM image of AA2024–AA1050/Al_2_O_3_ nanocomposite created by **a** 1, **b** 3, and **c** 6 ARB cycles and **d** SEM image of AA2024–AA1050 nanocomposite produced by the six ARB cycles

Figure 4a–c show the images taken by SEM from AA2024–AA1050/Al_2_O_3_ nanocomposites after 1, 3, and 6 cycles of ARB, respectively. Also, Fig. 4d shows the image of AA2024–AA1050 nanocomposite created by six ARB cycles. After an ARB cycle, the AA2024 layer was still continuous. Nevertheless, with more rolling cycles, the AA2024 layer was broken. A failure occurred in shear bands (at 45°). Due to the strain from rolling, the AA2024 layers stretched, and the fracture angle reduced from 45° to 20°–30°. After breaking the AA2024 layer, reducing the thickness of these layers during rolling was much less of a prediction.

**Fig. 5:**
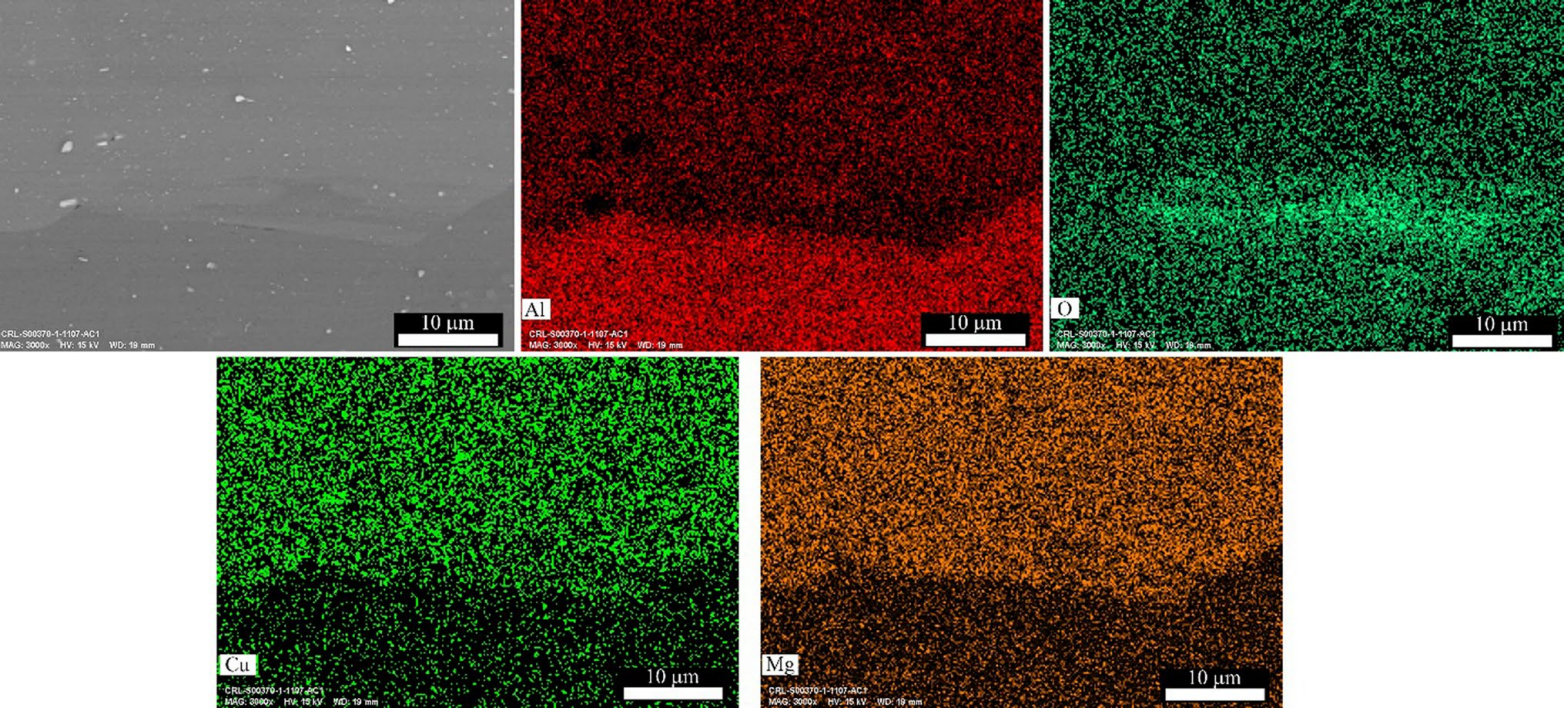
SEM image and EDS-MAP analysis of AA2024–AA1050/Al_2_O_3_ nanocomposite created with one cycle of the ARB process

**Fig. 6:**
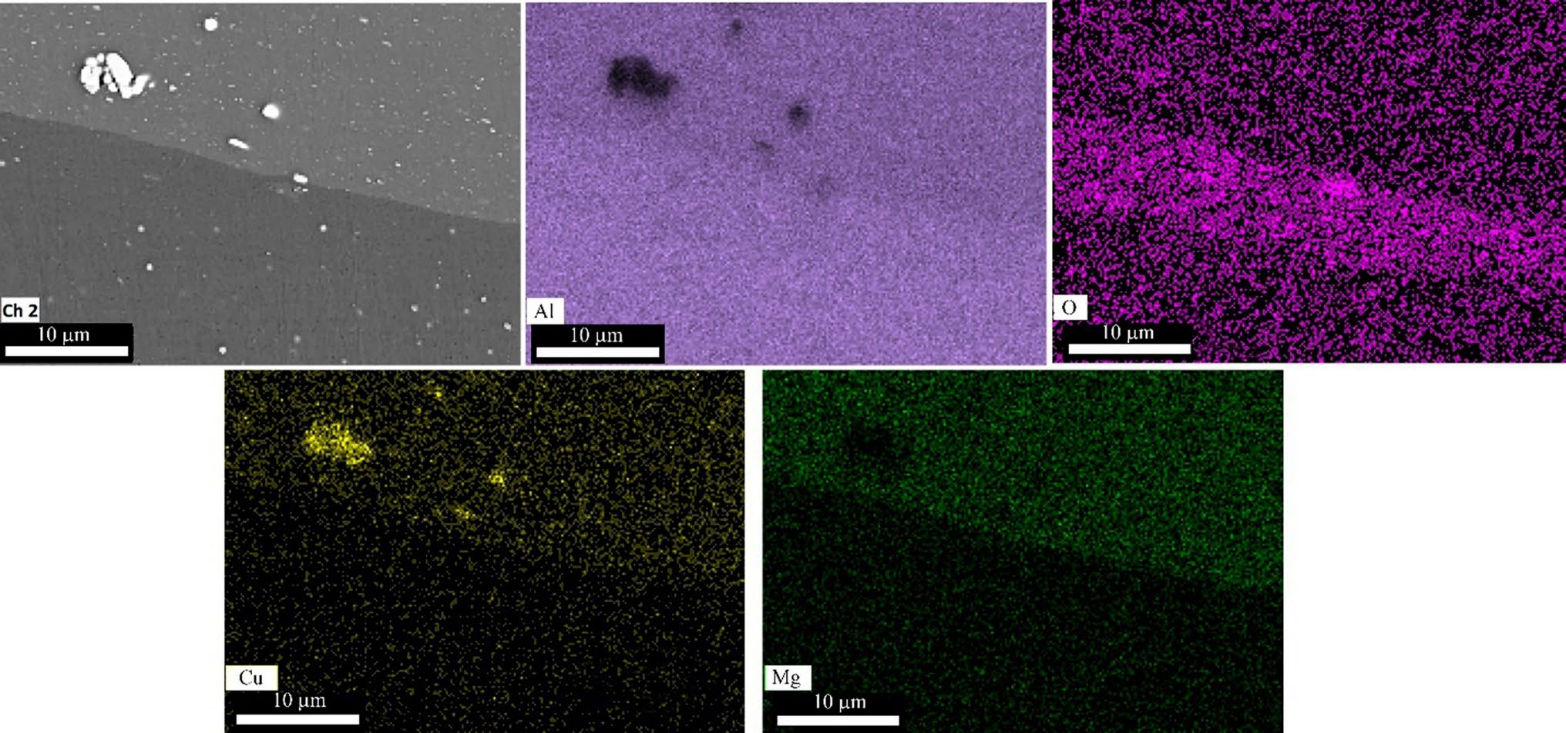
SEM image and EDS-MAP analysis of AA2024–AA1050/Al_2_O_3_ nanocomposite created with six cycle of the ARB process

The amount of use of alumina nanoparticles was meager. EDS-MAP analysis examined how these particles are dispersed in the composite. Figures 5 and 6 show the results of the EDS-MAP analysis. After the first cycle (Fig. 5), the aluminum oxide was between the AA2024 and AA1050 layers and was a dense layer with a 5 µm width. Furthermore, no cracks existed between the AA1050 and AA2024 layers and around alumina nanoparticles. With six cycles of ARB, alumina nanoparticles were not visible in the composite matrix (Fig. 6). EDS-MAP only showed the oxygen concentration uniformly, indicating the uniform distribution of alumina. Therefore, the distribution of alumina particles after 6 ARB cycles was perfect. Also, the border between AA2024 and AA1050 was smooth after six ARB cycles. The pressure applied by the rolling mill caused the existing scratches from brushing to disappear.

**Fig. 7:**
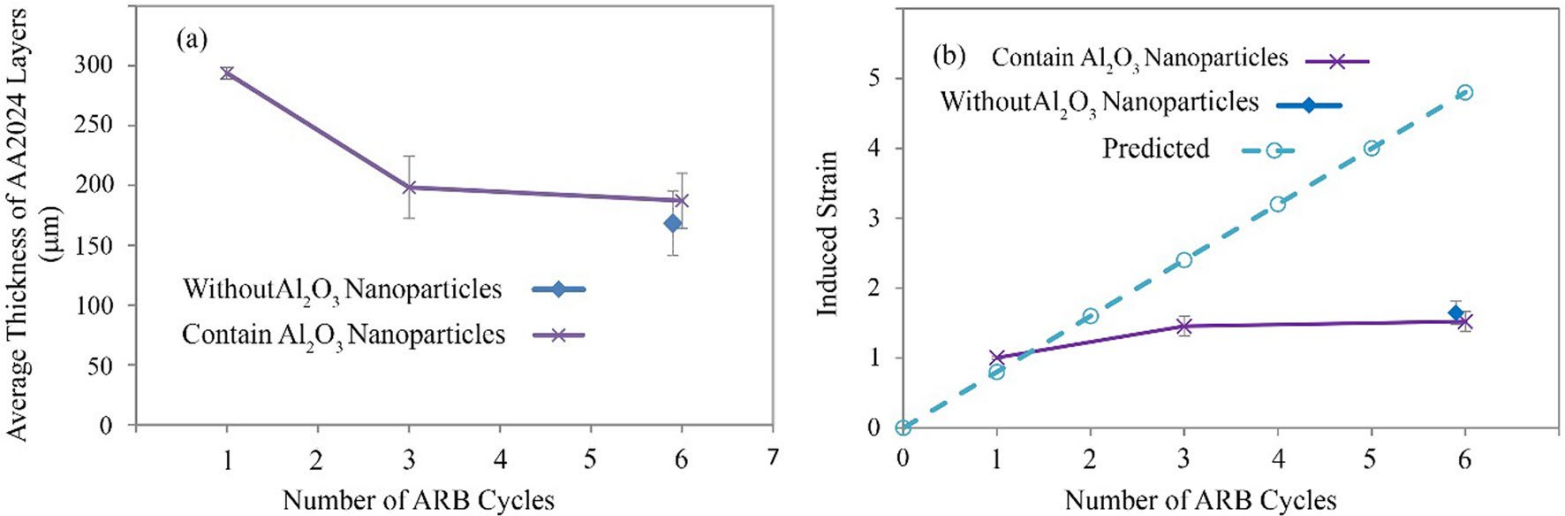
**a** Graph of ARB cycles-the thickness of AA2024 parts and **b** graph of ARB cycles-applied strain to AA2024 parts

According to Fig. 4, performing various ARB cycles caused the thickness of the AA2024 layers to be reduced. In order to examine more detail, ImageJ software measured the thickness after each cycle. For this purpose, ImageJ software examined the picture of more than 50 pieces of AA2024. Figure 7a and b show AA2024 thickness and applied strain to the layers of AA2024 after each rolling cycle, respectively. After the first cycle of ARB, the applied strain and the thickness decrease were equal to the prediction.

Nevertheless, as ARB cycles increased (due to the AA2024 layer break), the applied strain and the thickness decrease were much less than predicted. After six ARB cycles, there was no distinction between the thickness of the AA2024 parts in the AA2024–AA1050 nanocomposite containing alumina nanoparticles and the composite without Al_2_O_3_ particles. However, the length of the AA2024 layers in the nanocomposite lacking Al_2_O_3_ nanoparticles was taller.

**Fig. 8:**
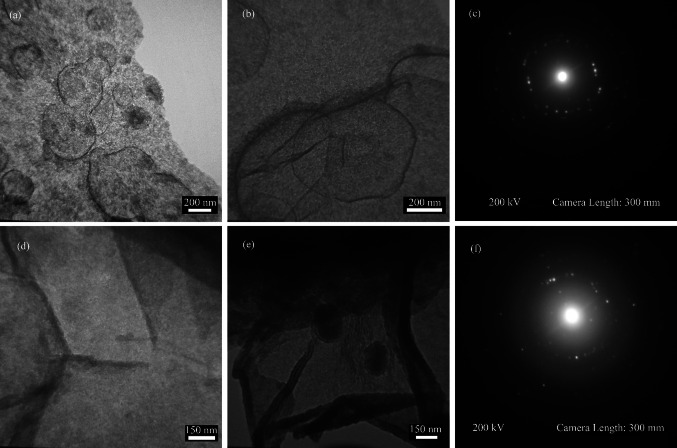
TEM image of **a** AA1050 layer, **b** AA2024 layer and **c** SAD pattern of AA2024–AA1050/Al_2_O_3_ nanocomposite and TEM image of **d** AA1050 layer **e** AA2024 layer and **f** SAD pattern of AA2024–AA1050 nanocomposite

Figure 8 shows the TEM image of six cycles ARB-ed the AA2024–AA1050 and AA2024–AA1050/Al_2_O_3_ nanocomposite. It shows the equiaxed grains in the TEM image of the AA2024–AA1050/Al_2_O_3_ nanocomposite. Aluminum grains in the AA1050 layers (about 200 nm diameter) were smaller than the AA2024 layers (about 500 nm diameter). Nevertheless, in the AA2024–AA1050 composite, the grains were multifaceted and stretched. Also, the grains were bigger than the same alloys in the AA2024–AA1050/Al_2_O_3_ composite.

Figure 8c and f show the SAD pattern of AA2024–AA1050/Al_2_O_3_ and AA2024–AA1050 nanocomposites, respectively. The diffraction pattern's point mode indicates the microstructure's crystalline nature. The number of points indicates the presence of tiny grains, like what we see in the TEM images [14, 29]. Also, AA2024–AA1050/Al_2_O_3_ nanocomposite points are more than AA2024–AA1050 in the SAD patterns, indicating that the AA2024–AA1050/Al_2_O_3_ microstructure is finer than AA2024–AA1050.

### Mechanical properties of AA2024–AA1050 nanocomposites produced by ARB process

**Fig. 9:**
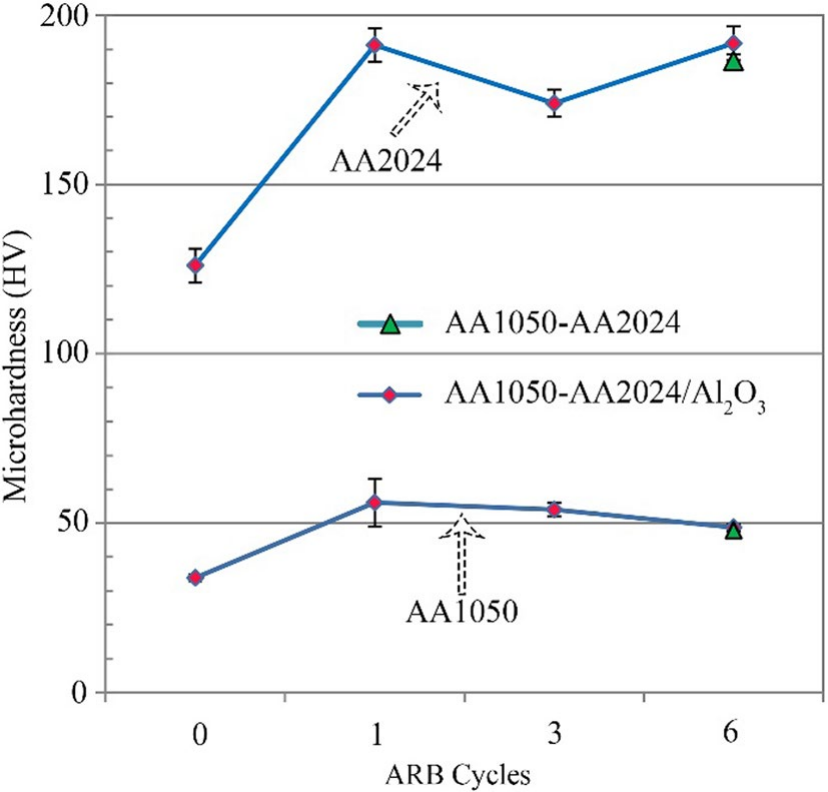
The microhardness graphs of AA2024–AA1050 nanocomposites with and without Al_2_O_3_ nanoparticles after various cycles of ARB

Figure 9 shows the obtained mean microhardness results after different ARB cycles. After the initial ARB cycle, the microhardness of the AA2024 and AA1050 layers incremented by 66% and 52% in the AA2024–AA1050/Al_2_O_3_ nanocomposite, respectively.

After six ARB cycles, the microhardness of the 1050 aluminum alloy was reduced by 13% compared to the composite created by an ARB cycle in the AA2024–AA1050/Al_2_O_3_ nanocomposite. Nevertheless, there was no significant difference in the microhardness of the AA2024 layers. The microhardness of the AA1050 and AA2024 layers in the AA2024–AA1050 nanocomposite was similar to the AA2024–AA1050/Al_2_O_3_ nanocomposite.

**Fig. 10:**
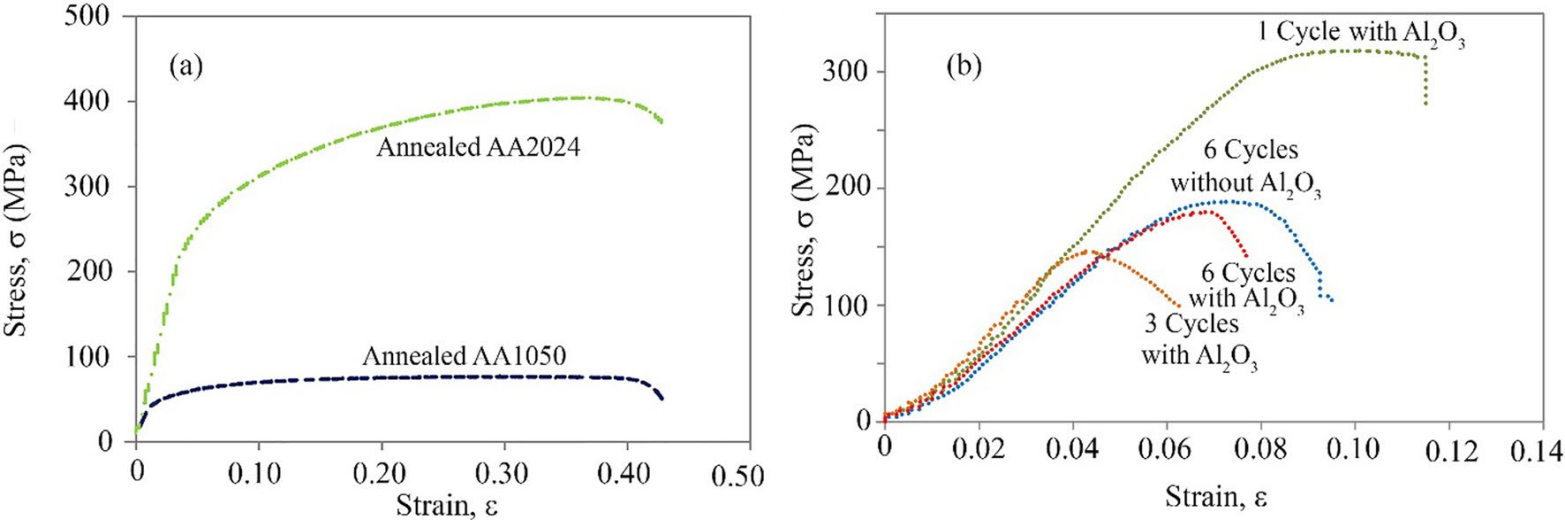
The strain–stress diagrams of **a** annealed AA1050 and AA2024 and **b** AA2024–AA1050 nanocomposites with and without Al_2_O_3_ after different ARB cycles

Figure 10 shows the tensile strength test results of the annealed 1050 and 2024 aluminum alloys and the AA2024–AA1050 and AA2024–AA1050/Al_2_O_3_ nanocomposites measured after different ARB cycles. After the first ARB cycle, the tensile strength of AA2024–AA1050/Al_2_O_3_ compared to annealed AA1050 increased by more than four times. Nevertheless, the composite tensile strength decreased by about 20% compared to the AA2024. After 3 ARB cycles, tensile strength declined sharply. However, with 6 ARB cycles, tensile strength increased by 23% compared to 3 ARB cycles.

The total elongation was significantly reduced in the early ARB cycles compared to annealed AA1050 and annealed AA2024. However, the elongation of 6 cycles ARB-ed composites increased slightly compared to 3 cycles ARB-ed composites. After 6 ARB cycles, the total elongation and tensile strength of AA2024–AA1050/Al_2_O_3_ nanocomposite were less than the AA2024–AA1050 nanocomposite.

**Fig. 11:**
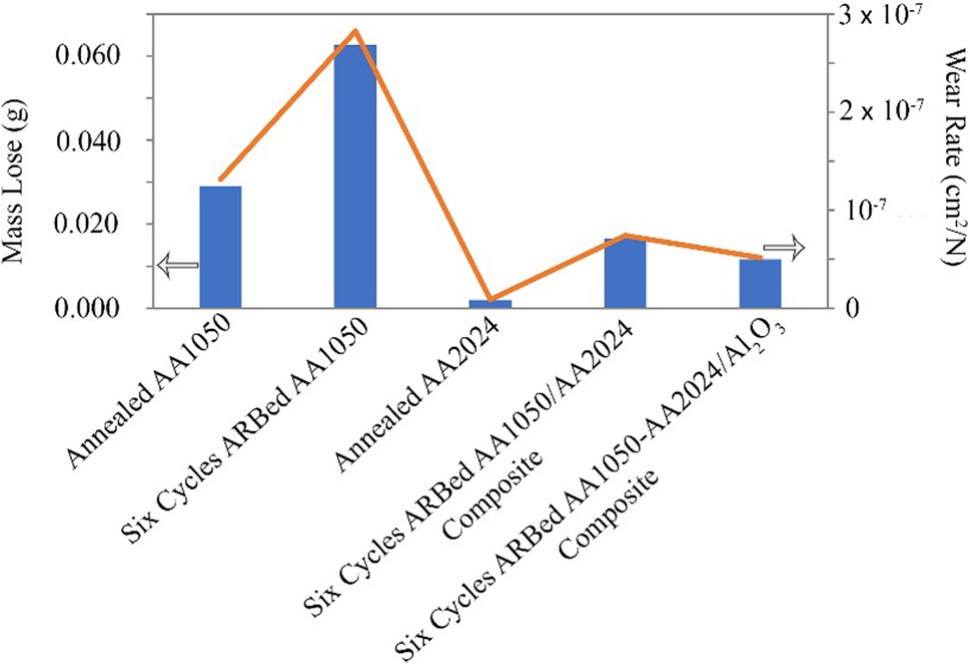
Weight loss and wear rate during Pin-on-Disc test for annealed AA2024 and AA1050, six cycles ARB-ed AA1050 and AA2024–AA1050 nanocomposites with and without Al_2_O_3_ nanoparticles produced by the six cycles of ARB method

Researchers perform the Pin-on-Disc wearing test to evaluate the wear resistance of the created composites. We performed the wearing test on the annealed AA1050 and AA2024 and composites of AA2024–AA1050/Al_2_O_3_ and AA2024–AA1050 after six ARB cycles. Also, for better comparison, AA1050 after six cycles ARB was tested. Figure 11 shows the results of the wearing test. This study used Eq. 1 to calculate the wear rate shown in Fig. 11.

1 $$Wear Rate=m/(\rho.F.L)$$

In Eq. 1, F, L, m, and ρ are the force of pin (N), abrasion test length (cm), reduced mass during wearing (g), and density (g cm^−3^), respectively.

The wear resistance of the AA2024 was 14.5 times more than the AA1050. Also, with six ARB cycles on the AA1050, the mass loss increased more than twice as much. This result means that wear resistance was reduced to less than half. Making the AA2024–AA1050 composite improved wear resistance by 43% compared to the annealed AA1050 and 74% compared to the six cycles ARB-ed AA1050. Nevertheless, the 2024 aluminum alloy wear resistance was eight times more than the AA2024–AA1050 composite. Using 0.005 vol.% alumina nanoparticles in the composite and creating AA2024–AA1050/Al_2_O_3_ nanocomposite improved 30% wear resistance compared to AA2024–AA1050 composite.

**Fig. 12:**
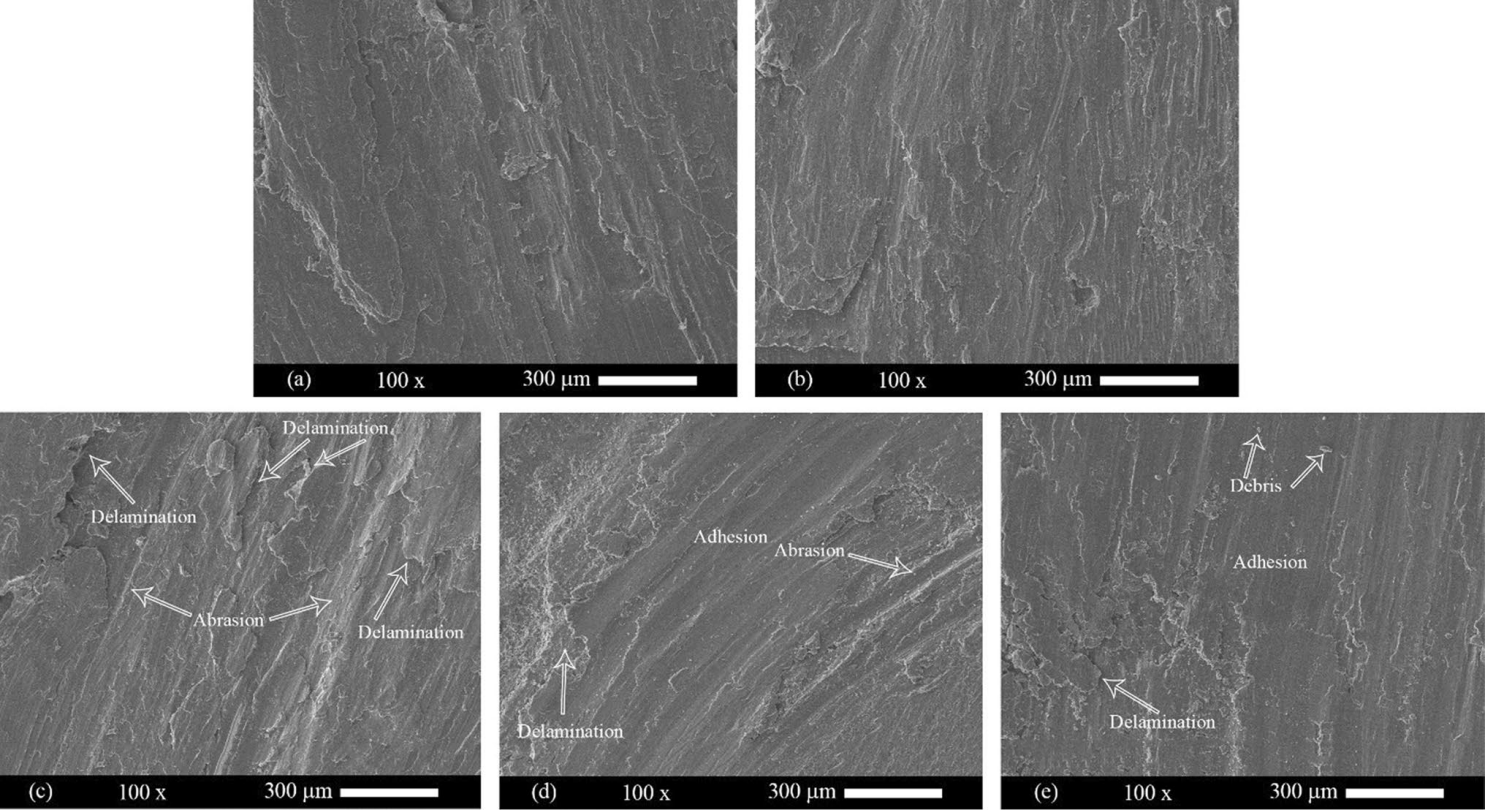
SEM images from worn surfaces of **a** Annealed AA1050, **b** Annealed AA2024, **c** Six cycles ARB-ed AA1050, **d** AA2024–AA1050 nanocomposite, and **e** AA2024–AA1050/Al_2_O_3_ nanocomposite produced by six ARB cycles

Figure 12 shows the surface of the worn samples. The worn surface of the annealed sheets was smooth and had narrow furrows (Fig. 12). This indicates the wear mechanisms of adhesive and abrasive, respectively. In some areas, crusts were observed. Crusts indicate the mechanism of delamination. The mechanism of delamination has the highest wear rate. Also, with the increase in the depth of the grooves, it can be concluded that wearing has increased. Six cycles of ARB significantly increased the delamination mechanism and depth of the furrows in the 1050 aluminum alloy. In the AA2024–AA1050 nanocomposite produced by six ARB cycles, the depth of grooves and delamination mechanism was decreased compared to the ARB-ed AA1050. In the AA2024–AA1050/Al_2_O_3_ nanocomposite, the depth of the grooves and delamination areas was much less than the composite without Al_2_O_3_. A smoother surface indicates an increase in surface wear resistance.

**Fig. 13:**
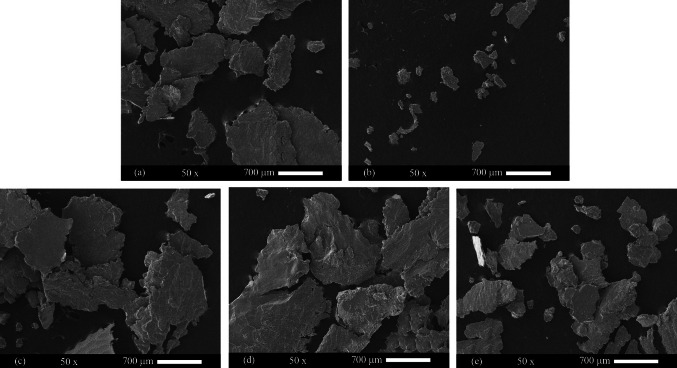
The debris of Pin-on-Disc test SEM images of **a** Annealed AA1050, **b** Annealed AA2024, **c** Six cycles ARB-ed AA1050, **d** AA2024–AA1050 composite, and **e** AA2024–AA1050/Al_2_O_3_ nanocomposite produced by six ARB cycles

Figure 13 shows the debris from the wearing test. These images show that the debris of AA2024 is trace. Also, spring-shaped debris was seen, indicating the abrasion mechanism. The visibility of the shells indicates the mechanisms of delamination and adhesion mechanisms. The size and volume of the debris in the six cycles ARB-ed AA1050 and the AA2024–AA1050 nanocomposite was high. However, the size and volume of the debris in the AA2024–AA1050/Al_2_O_3_ nanocomposite were less than in other ARB-ed samples. Decreasing the debris volume confirmed the wear resistance improvement by adding 0.005% alumina to the AA2024–AA1050 nanocomposite.

### Effect of the aging process on the AA2024–AA1050/Al_2_O_3_ nanocomposite produced by ARB

The aging process was performed at different temperatures and times to evaluate the effect of the aging process on the mechanical properties of the AA2024–AA1050/Al_2_O_3_ nanocomposite. According to the tests, one can obtain the mechanisms of the impact of the aging process on the AA2024–AA1050/Al_2_O_3_ nanocomposite.

**Fig. 14:**
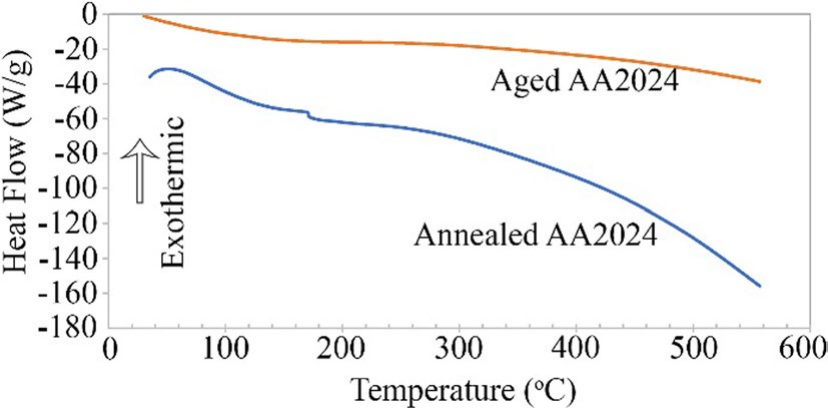
Patterns of the DSC analysis for the AA2024 sheet have been annealed and after the aging process at 190 °C for 30 min

In order to ensure the annealing process for AA2024, DSC analysis was performed after the annealing and after an aging process. The aging process was done at 190 °C for 30 min on the annealed sheet. Figure 14 shows the DSC analysis diagrams for the annealed aged AA2024. Analysis was performed at 10 °C/min speed and in the argon atmosphere. There are many differences between these two diagrams. In the diagram of annealed AA2024, there is a peak at 50 °C. According to scientific sources, this courier is related to the formation of GPB [9, 30]. At about 175 °C, there is also a peak. This change can be due to the creation of S' precipitates. Neither of these two cases was in the aged AA2024 DSC diagram. Therefore, it can be claimed that GPB and S′ (Al_2_CuMg) precipitates were created in the aged AA2024 sheets.

**Fig. 15:**
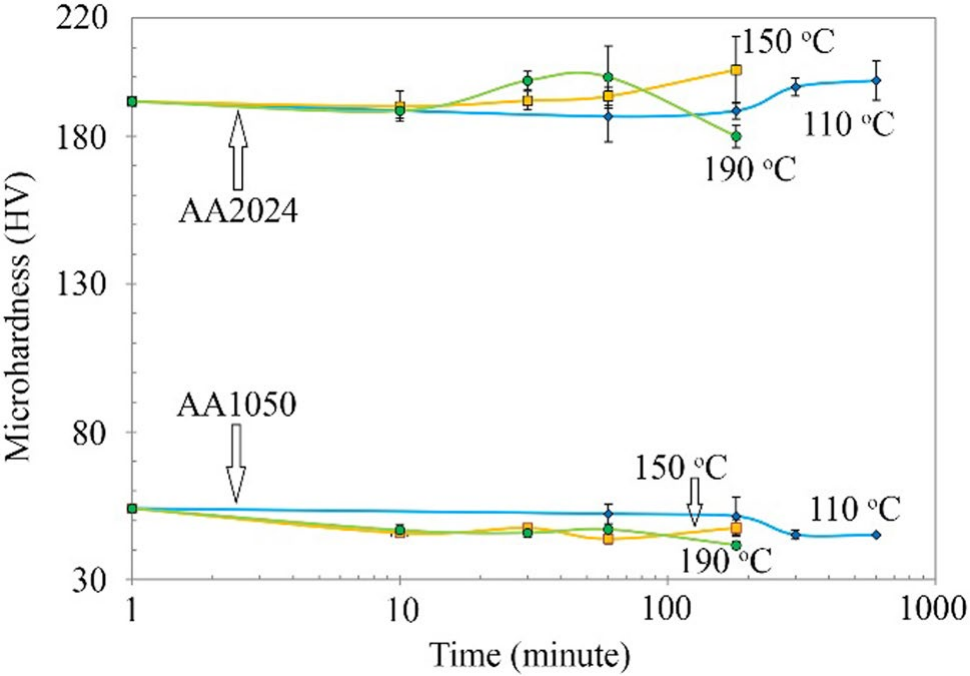
The microhardness test results for AA2024–AA1050/Al_2_O_3_ nanocomposites after keeping at temperatures of 110, 150, and 190 °C for various times

The AA2024–AA1050/Al_2_O_3_ nanocomposite aged at different temperatures and times. Figure 15 shows the results of the microhardness test for aged composites. The microhardness of AA2024 in all samples was about four times that of AA1050. Furthermore, aging at 110, 150, and 190 °C reduced the microhardness of the AA1050 layers by 8.9, 10.25, and 12.39 HV, respectively. However, it was harder than before ARB. Reducing the microhardness is due to recovery at the furnace temperature.

Further, the AA2024 layers showed a different trend. The microhardness of these layers in the furnace increased at 190 °C and then decreased. However, in the furnace at 110 °C and 150 °C, they increased until the scheduled period for aging. Due to the scientific background of Al–Cu and AA2024 alloys, microhardness must reduce at more aging times with these temperatures [14]. According to the data, the maximum microhardness of the aged AA2024 layers at 110, 150, and 190 °C were 7.13, 10.75, and 8.27 HV more than AA2020–AA2024/Al_2_O_3_ before aging. The microhardness increased due to the precipitations in the AA2024 layers. Due to the lack of a drop in microhardness graphs at 110 and 150 °C, microhardness may increase in these temperatures in more time.

**Fig. 16:**
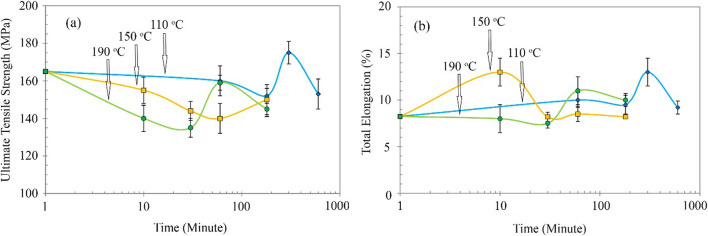
**a** Ultimate tensile strength (UTS), and **b** total elongation for AA2024–AA1050/Al_2_O_3_ nanocomposites at different temperatures and times

We performed the tensile strength test after aging to study the tensile strength and ductility of the aged AA2024–AA1050/Al_2_O_3_ nanocomposites. Figure 16 shows the UTS and total elongation diagrams harvested from these tests. With the aging, the tensile strength gradually decreased at temperatures 110, 150, and 190 °C, then increased and eventually reduced again. The minimum tensile strength in aged composites at 190 °C was lower than other composites. Also, the highest tensile strength belonged to the composite aged 110 °C.

The minimum “total elongation” (7.5%) belonged to the composite aged at 190 °C by comparing the elongation change in different temperatures and times. In contrast, the highest total elongation (13%) belonged to samples at 110 °C and 150 °C. However, the total elongation had an oscillation. So, it first decreased, then increased, and reduced again. This oscillation occurred faster in aging at 150 and 190 °C due to higher temperatures than 110 °C.

**Fig. 17:**
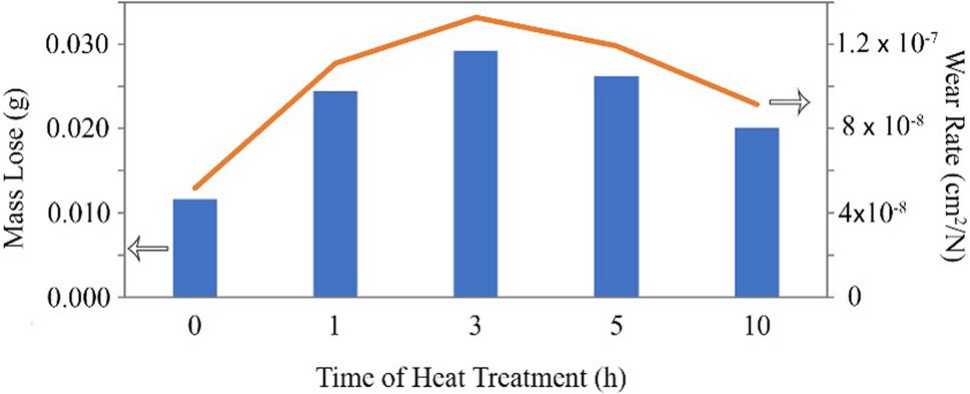
Reduced mass during Pin-on-Disc test and wear rate of AA2024–AA1050/Al_2_O_3_ aged at 110 °C

The AA2024–AA1050/Al_2_O_3_ nanocomposites aged at 110 °C had better tensile strength and elongation than other temperatures. Therefore, we selected this temperature as the optimal temperature. Figure 17 shows the Pin-on-Disc test results for aged composites at this temperature. According to Fig. 17, the aging process reduced the wear resistance by up to 3 h. Nevertheless, with the increased aging time to 10 h, the wear resistance increased by 31% compared to the composite of 3 h.

**Fig. 18:**
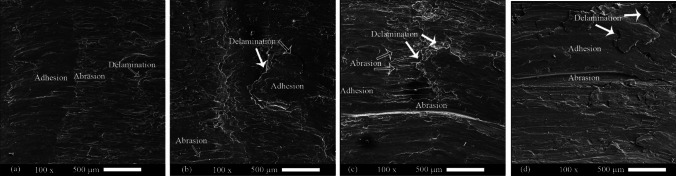
SEM images of worn surfaces of AA2024–AA1050/Al_2_O_3_ nanocomposites aged at 110 °C for **a** 1 h, **b** 3 h, **c** 5 h, and **d** 10 h

Figure 18 shows the SEM images of the worn surfaces of the AA2024–AA1050/Al_2_O_3_ nanocomposites aged at 110 °C. In the one-hour aged composite, the adhesive and abrasion mechanisms were dominant. The delamination mechanism expanded in composites aged 3 and 5 h, and the abrasions were deeper than others. However, the worn surface of 10 h aged nanocomposite showed fewer scratches and crusts.

**Fig. 19:**
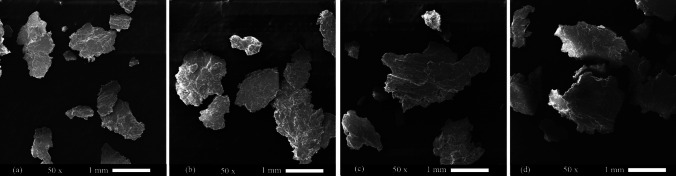
SEM image of worn debris of AA2024–AA1050/Al_2_O_3_ nanocomposites that aged for **a** 1 h, **b** 3 h, **c** 5 h, and **d** 10 h at 110 °C

Figure 19 shows the SEM images of the debris from wearing the surfaces of aged AA2024–AA1050/Al_2_O_3_ nanocomposites. The debris size of the one-hour-aged composite was less than that of other composites. Furthermore, according to the increase in wearing in Fig. 17, the debris size of the composites aged for 3 and 5 h increased. Nevertheless, by 10 h of aging, the debris size was decreased compared to 3 and 5 h of aging.

## Discussion

### Microstructures of composites that created by ARB

The aluminum alloy of 2024 is four times harder than the AA1050. For this reason, the AA2024 has a lower strain speed than the AA1050. Therefore, the higher hardness of AA2024 caused the crush of AA2024 layers in the AA1050 (Fig. 4). The high strain at each rolling cycle caused deformation limited through slip systems. Therefore, the AA2024 layers are broken in the shear band (45°) where shear stress is maximum. Due to the pressure applied to the AA2024 layers, the angle of 45° was reduced to the AA2024 pieces and reached about 30°. Also, the breakdown of the AA2024 layers caused less stress applied to these layers. As a result, most of the stress flowed in the AA1050 continuous matrix. Thus, the thickness of the 2024 aluminum alloy pieces was more than the calculated value (Figs. 4 and 7).

Performing each cycle of ARB causes too much strain on the composite sheet. Therefore, the sheets' length doubled, and the distance between particles increased by the applied strain. A few steps to increase the length and to put the sheets together caused the uniform distribution of Al_2_O_3_ nanoparticles after six rolling cycles (Figs. 5 and 6).

As mentioned, rolling causes much strain applied to the material. This strain causes work hardening and creates a high density of dislocations. In 2024 layers, intrinsic precipitates such as T-phase (Al_20_Cu_2_Mn_3_), Al_77_Cu_10_Fe_7_Mn_5_Si_2_, Al_79_Cu_13_Fe_5_Mn_2_Mg_0.7_Si_0.2_, and Al_65_Cu_17_Mg_18_ also play a significant role [23, 31]. During rolling, differences in the flow of the metal matrix and precipitates cause dislocations at the boundary between the precipitates and the metal matrix (Fig. 4). During successive rolling, dislocations are formed as sub-grained structures and cause continuous dynamics recrystallization [10, 15, 32]. In this way, ARB creates a UFG structure (Fig. 8).

AA1050 is much softer than AA2024. For this reason, alumina nanoparticles sank in AA1050. They thus had more impact on it (Fig. 6). In this way, with the excellent distribution and proper connection of these particles to the metal matrix, sufficient interaction occurred during rolling and caused the formation of a high density of dislocations at the boundaries of the reinforcement particles and the metal matrix, which played an essential role in creating of UFG. Also, these particles, by sinking in AA1050, increased the wear resistance of these layers.

According to the discussion of strain in the 2024 and 1050 aluminum alloy layers, the smaller grains in the AA1050 layers found in the TEM images were expected.

Alumina nanoparticles in AA2024–AA1050/Al_2_O_3_ nanocomposite made aluminum grains uniaxial. Uniaxial grains in rolled aluminum were unlike the microstructure of ARB-ed aluminum [3, 12]. In ARB-ed, only continuous dynamic recovery occurs due to high staking fault energy (SFE) and then easy recovery of dislocations. For this reason, the rolling draws the grains in the rolling direction. Recovery is not easily performed in metals with low SFE, such as copper. Therefore, there are sufficient dislocations to create a homogeneous structure. As a result, discontinuous dynamics re-crystallization occurs, and the grains are equiaxed [33, 34].

In AA2024–AA1050/Al_2_O_3_ nanocomposite, four strains followed:

- The strain applied by the thickness reduction
- Strain due to friction of rollers and aluminum sheets
- The strain between the layers of AA2024 and AA1050 due to the difference in the strain behavior
- The strain created in aluminum close to the surface of Al_2_O_3_ nanoparticles

The fragmentation of the AA2024 layers increased their contact with the AA1050. Also, alumina nanoparticles had a higher surface area than micrometer alumina particles and thus significantly affected much lower value (here 0.005 vol.%). Generally, the difference between structural characteristics and differences in strain behavior of aluminum and ceramic particles causes a high density of dislocation between aluminum and reinforcing particles by strain.

There were four strains mentioned above in another research. However, their sum at the same time was scrutinized only in the project. In other research, aluminum grains were stretched in the rolling direction without a strain between the layers or caused by reinforcing particles.

Simultaneously using reinforcing particles, creating a layered composite, and rolling without lubricant caused an increase in the dislocation density in the AA2024–AA1050/Al_2_O_3_ nanocomposite. Therefore, UFG microstructures with often 200 nm diameter in the AA1050 and grains with a maximum diameter of 500 nm were created in the AA2024 layers.

### Mechanical properties of AA2024–AA1050 composites created by the ARB method

The rolling pressure at the rolling cycle creates a high density of dislocations. These dislocations cause work hardening and increase hardness. Also, the created work hardening increases the tensile strength. The dislocation arrangement by performing more ARB cycles makes sub-grain boundary structures by performing more ARB cycles. Hence, UFG creation reduces the density of the dislocation and the hardness. However, the dislocation density and work hardening are much more than the initial sheets [10, 19].

The mentioned items in the above paragraphs were in the AA2024–AA1050/Al_2_O_3_ composite. Nevertheless, the microhardness of the AA2024 layers did not decrease, unlike the AA1050 (Fig. 9). The difference between the strain properties of AA2024 with the AA1050 matrix and the various precipitates in the AA2024 layers, like T-phase, GPB, S′, Al_77_Cu_10_Fe_7_Mn_5_Si_2_, Al_79_Cu_13_Fe_5_Mn_2_Mg_0.7_Si_0.2_, and Al_65_Cu_17_Mg_18_ precipitates [9, 30, 35], made more dislocation and ongoing work hardening in these layers.

Naturally, the tensile strength of AA2024 is higher than AA1050 due to the soluble elements of copper and magnesium and various precipitates. About AA2024–AA1050 composites, by performing an ARB cycle, the AA2024 layer was continuous. For this reason, the 2024 and 1050 aluminum alloy layers tolerate the tensile stress. Therefore, the composite had high tensile strength and elongation (Fig. 10). By performing more ARB cycles, the AA2024 layers were fragmented. Thus, the continuous layers of the 1050 aluminum alloy were the primary origin of tensile strength. Therefore, tensile strength and elongation decreased. In particular, the sharp edges of the AA2024 pieces caused stress concentration.

With more ARB cycles and the creation of the UFG structure, the tensile strength was increased compared to the second cycle. Creating a UFG structure increases the density of the boundary. As such, it shows more resistance to cracking. Nevertheless, the presence of ceramic particles due to the incoherency of the particles in the metal matrix causes the concentration of stress and reduces tensile strength [10, 19, 28]. Therefore, after six ARB cycles, the total elongation and tensile strength of AA2024–AA1050/Al_2_O_3_ nanocomposite was less than that of the AA2024–AA1050 nanocomposite. However, the opposite was the case for wear resistance.

The following three general mechanisms are for wearing [4, 36]:

- Abrasion
- Adhesion
- Delamination

The mechanism of delamination has the most mass harvesting from the surface. Passing the abrasive pin by applying pressure causes fatigue below the surface and overrides the pieces from the surface. Due to the layered structure of the ARB-ed metal, the ARB process increases the delamination mechanism and reduces wear resistance [37, 38]. The connection between the ARB-ed metal layers is mechanical and not metallurgical. Therefore, the wear resistance of the ARB-ed AA1050 was much less than the annealed AA1050 (Fig. 11).

Due to soluble copper and magnesium in aluminum and different precipitates, the AA2024 wear resistance is much better than the AA1050. Also, ceramic particles reduce the delamination mechanism and the depth of grooves and increase wear resistance because of their high hardness [4, 39, 40]. The hard particles between the metal matrix and the wearing pins cause to reduce the wear rate. The operation of ceramic particles at wearing is contrary to tensile strength. Therefore, the stress concentration between the reinforcement particles and the metal matrix reduces the tensile strength. Nevertheless, the force in wear is pressure. Thus, reinforcing particles increase the resistance to wear.

**Fig. 20:**
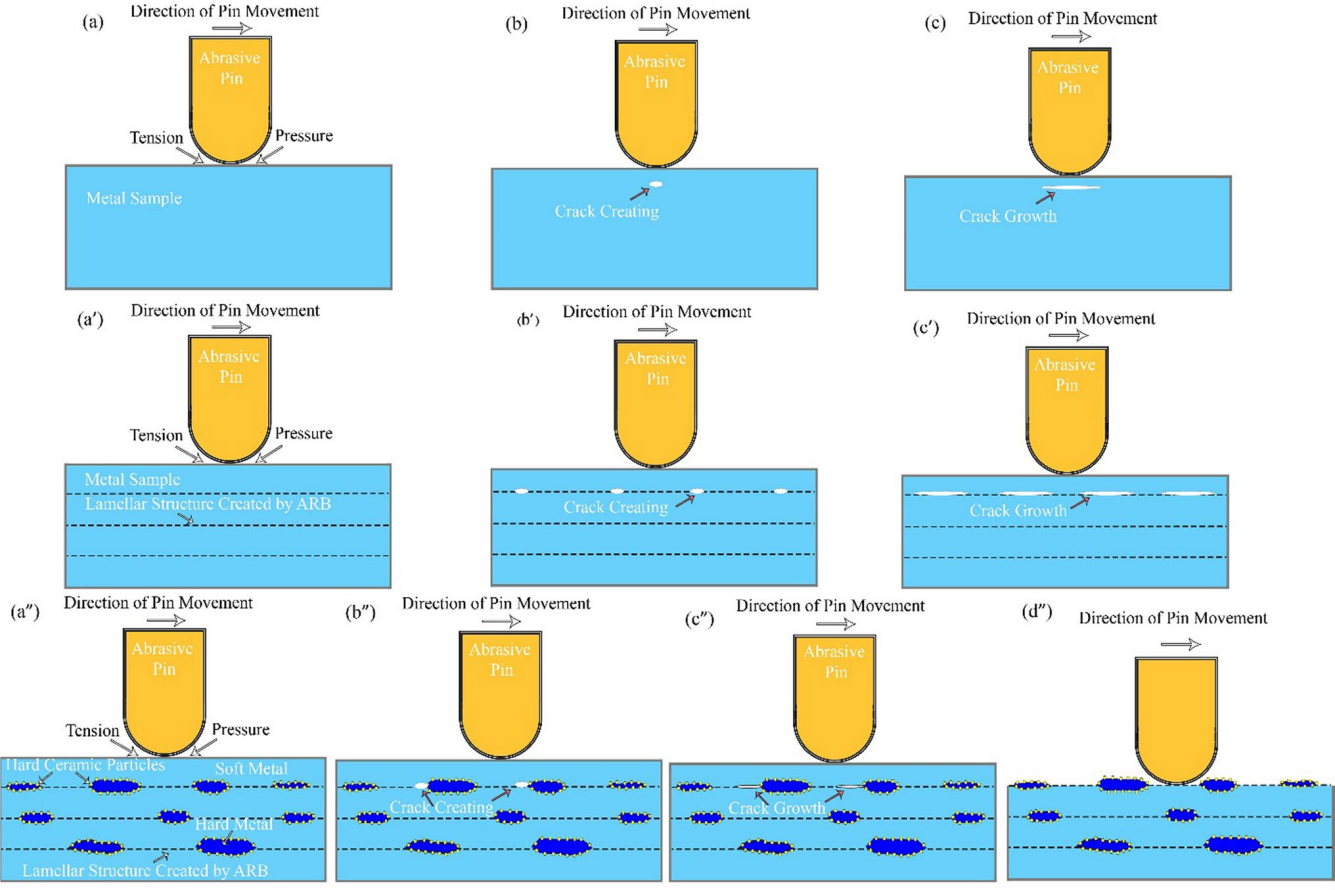
**a** Starting the wear test on integrated metal, **b** to create fatigue crack in the layer below the pin, **c** Expanding the crack due to the rotation of the pin and the applying of force, **a′** Starting the wear test on ARB-ed metal, **b′** to create fatigue crack in the ARB-ed metal sheet, **c′** expansion of cracking in ARB-ed metal, **a″** Starting wearing test on the layered composite created by ARB including reinforcing particles, **b″** Creating fatigue crack in composite, **c″** Expanding the cracks in the composite due to the rotation of the pin and the use of force

Creating a lamellar composite between 2024 and 1050 aluminum alloys causes an increase in wear resistance compared to AA1050. Improvement of wear resistance is due to the higher wear resistance of AA2024 compared to AA1050 (Fig. 11). Also, with alumina particles, the wear resistance increased due to alumina particles' high hardness. Furthermore, the high specific surface area of the Al_2_O_3_ nanoparticles compared to micrometer particles, a small amount of these nanoparticles (0.005 vol.%) significantly increased wear resistance. Figure 20 shows all the mentioned content and mechanisms presented in the above and this paragraph.

It mentioned ARB uniformly distributed alumina nanoparticles after six cycles between 1050 and 2024 layers. Also, these particles sank more in the soft layers of 1050. In this way, particles sinking into 1050 layers, proper distribution and connection with the metal matrix of these particles increased the wear resistance of the entire surface of 1050 layers and, as a result, the entire composite.

### Effect of the aging process on the AA2024–AA1050/Al_2_O_3_ nanocomposite produced by ARB

The heating has been recovered and reduces the hardness of pure metals. However, in alloys with precipitation capable, heating also causes the development of the precipitates (aging process). Precipitates in coherent and semi-coherent states increase hardness. Nevertheless, in the over-aged mode, the hardness is reduced due to emptying the metal matrix from the soluble elements. Also, higher temperatures cause processes to occur faster because higher temperatures increase penetration. As such, recovery or precipitation growth occurs faster than lower temperatures [8, 9, 30].

The abovementioned cases were done by aging AA2024–AA1050/Al_2_O_3_ (Fig. 15). The primary precipitate in AA2024 is Al_2_CuMg. The AA1050 is not capable of an aging process. For this reason, its hardness was reduced by furnace heat. Aging of AA2024 typically takes about 8 h at 190 °C to get the highest hardness [30, 35]. However, in this project, due to ARB, it reached its maximum microhardness at 1 h at 190 °C (Fig. 15). ARB creates a high density of dislocations and new paths for penetrating alloying elements. Thus, the ARB process increased the speed of the aging process.

Despite the relatively high temperature and the long time of the aging process, the microhardness of the AA1050 did not return to the amount of annealed aluminum (Figs. 9 and 15). Generally, the hardness in the AA1050 depends on the two factors of work hardening and grain size. The aging process reduced the work hardening. However, the temperatures were lower than aluminum recrystallization (more than 230 °C) [41, 42]. For this reason, a decrease in microhardness occurred due to recovery.

Nevertheless, it did not decrease hardness due to recrystallization. As a result, the UFG structure and some work hardening are still residual and have increased hardness. The non-increase in total elongation and the decline in tensile strength to the annealed AA1050 is a reason (Fig. 16).

As mentioned, heat treatment causes recovery and reduces work hardening. Decreasing work hardening was more evident in the AA1050, which could not precipitation. Hence, all three categories of the aging process reduced the tensile strength of the composite (Fig. 16). Furthermore, non-metallurgical bonds between the AA1050 layers and the AA2024 pieces can cause stress concentration and cracking. However, with more time in the aging process, creating precipitations, and improving the connection between AA1050 and AA2024, tensile strength increased (Fig. 16). The better connection between AA1050 and AA2024 reduced stress concentration and, as a result, total elongation increased.

As the time of the aging process increases, large coarse precipitates are created [28, 30, 35]. The boundary between incoherent precipitates and the metal matrix is a place for stress concentration. Therefore, incoherent precipitates reduce tensile strength. This phenomenon was well observed in AA2024–AA1050/Al_2_O_3_ composite aged at 150 and 190 °C in 3 and 5 h of aging (Fig. 16). However, the heat intensity in aging was lower at 110 °C. Therefore, less recovery occurred in the AA1050, and precipitations occurred in the AA2024 layers in a more balanced mode. Therefore, the tensile strength and total elongation of the AA2024–AA1050/Al_2_O_3_ nanocomposite was better at 110 °C.

Heat treatment reduced AA1050's microhardness (Fig. 15). The decrease in the microhardness of AA1050 reduced its wear resistance. Therefore, because the AA1050 constituted about 66% of the composite volume of AA2024–AA1050/Al_2_O_3_, the wear resistance of this composite was reduced. Thus, a decrease of wear resistance continued for three hours in the furnace at 110 °C (Fig. 17). While at more time, the aging process done by more impact in the AA2024, which is why the wear resistance of the AA2024–AA1050/Al_2_O_3_ composite increased. The volume of the segregated shells and the depth of the scratches increased due to a decrease in composite wear resistance, and with the increased wear resistance, the volume of the disjunct shells and the depth of the scratches decreased (Figs. 18 and 19).

## Conclusion

This study created the AA2024–AA1050 and AA2024–AA1050/0.005 vol% Al_2_O_3_ nanocomposites by ARB. Along this path, the microstructure, mechanical, and aging properties were examined after the ARB process. Significant results are as follows:

1. Six cycles of ARB fragmented AA2024 and dispersed completely Al_2_O_3_ in AA1050 and created AA2024–AA1050/0.005 vol.% nanocomposite.
2. AA2024–AA1050/0.005 vol.% Al_2_O_3_ nanocomposite created by six ARB cycles had uniaxial grains by the size of about 200 nm and less than 500 nm for 1050 and 2024 aluminum alloys, respectively.
3. With six ARB cycles, due to the work hardening and grain refinement, the tensile strength of the AA2024–AA1050 nanocomposites increased by more than two times that of the annealed 1050 aluminum alloy.
4. Wear resistance improved by over 74% compared to the ARB-ed AA1050 with the creation of the AA2024–AA1050 nanocomposite.
5. Adding 0.005% of alumina nanoparticles to the AA2024–AA1050 composite improved its wear resistance by 30%.
6. The Aging process at 110 °C caused the tensile strength and total elongation to increase significantly and reduced the microhardness of AA1050 by a small amount.
7. Wear resistance of the AA2024–AA1050/0.005 vol.% Al_2_O_3_ with aging at 110 °C was first decreased due to the decrease in the microhardness of AA1050 and then increased due to the increased microhardness of AA2024.

## Data Availability

The authors confirm that the data supporting the findings of this study are available within the article.

## References

[1] Han P, Wang W, Liu Z, Zhang T, Liu Q, Guan X, Qiao K, Ye D, Cai J, Xie Y, Wang K (2022). Modification of cold-sprayed high-entropy alloy particles reinforced aluminum matrix composites via friction stir processing. J Alloys Compd.

[2] Wang SB, Liu ZR, Xia SL, Key J, Chen JH (2017). Tetragonal-prism-like Guinier–Preston–Bagaryatsky zones in an AlCuMg alloy. Mater Charact.

[3] Roghani H, Borhani E, Shams SAA, Lee CS, Jafarian HR (2022). Effect of concurrent accumulative roll bonding (ARB) process and various heat treatment on the microstructure, texture and mechanical properties of AA1050 sheets. J Mater Res Technol.

[4] Gholami MD, Salamat M, Hashemi R (2021). Study of mechanical properties and wear resistance of Al 1050/Brass (70/30)/Al 1050 composite sheets fabricated by the accumulative roll bonding process. J Manuf Process.

[5] Adachi H, Miyajima Y, Sato M, Tsuji N (2015). Evaluation of dislocation density for 1100 aluminum with different grain size during tensile deformation by using in-situ x-ray diffraction technique. Mater Trans.

[6] Sun L, Guo Y, Chen L, Zhao G (2021). Effects of solution and aging treatments on the microstructure and mechanical properties of cold rolled 2024 Al alloy sheet. J Mater Res Technol.

[7] Zheng R, Bhattacharjee T, Shibata A, Tsuji N, Ma C (2016). Effect of accumulative roll bonding (ARB) and subsequent aging on microstructure and mechanical properties of 2024 Al alloy. Mater Trans.

[8] Rodríguez-González P, Fernández-Abia AI, Castro-Sastre MA, Barreiro J (2020). Heat treatments for improved quality binder jetted molds for casting aluminum alloys. Addit Manuf.

[9] Chen X, Marioara CD, Andersen SJ, Friis J, Lervik A, Holmestad R, Kobayashi E (2021). Precipitation processes and structural evolutions of various GPB zones and two types of S phases in a cold-rolled Al–Mg–Cu alloy. Mater Des.

[10] Saito Y, Tsuji N, Utsunomiya H, Sakai T, Hong RG (1998). Ultra-fine grained bulk aluminum produced by accumulative roll-bonding (ARB) process. Scr Mater.

[11] Roghani H, Borhani E, Shams SAA, Lee CS, Jafarian HR (2021). On the microstructure, texture and mechanical properties through heat treatment in Al–CuO nanocomposite fabricated by accumulative roll bonding (ARB). Mater Sci Eng A.

[12] Yu H, Su L, Lu C, Tieu K, Li H, Li J, Godbole A, Kong C (2016). Enhanced mechanical properties of ARB-processed aluminum alloy 6061 sheets by subsequent asymmetric cryorolling and ageing. Mater Sci Eng A.

[13] Daneshvar F, Reihanian M, Gheisari K (2016). Al-based magnetic composites produced by accumulative roll bonding (ARB). Mater Sci Eng B.

[14] Tsuji N, Iwata T, Sato M, Fujimoto S, Minamino Y (2004). Aging behavior of ultrafine grained Al–2wt%Cu alloy severely deformed by accumulative roll bonding. Sci Technol Adv Mater.

[15] E. Borhani, B. Azad, A. Abdoos, Nano structures by severe plastic deformation (SPD) processes, chapter 9. In: Applied Mathematical Models and Experimental Approaches in Chemical Science, pp. 101–122. Apple Academic Press; 2016. 10.1201/9781315366203-10

[16] Arigela VG, Palukuri NR, Singh D, Kolli SK, Jayaganthan R, Chekhonin P, Scharnweber J, Skrotzki W (2019). Evolution of microstructure and mechanical properties in 2014 and 6063 similar and dissimilar aluminium alloy laminates produced by accumulative roll bonding. J Alloys Compd.

[17] Ye N, Ren X (2020). Microstructure and texture evolution of aluminum in the Al-Nb/Ti/Ni composite fabricated by the ARB process. J Chem.

[18] Chen Y, Nie J, Wang F, Yang H, Wu C, Liu X, Zhao Y (2020). Revealing hetero-deformation induced (HDI) stress strengthening effect in laminated Al-(TiB2+TiC)p/6063 composites prepared by accumulative roll bonding. J Alloys Compd.

[19] Roghani H, Borhani E, Jafarian HR (2021). Effect of a trace amount addition of CuO on aluminum sheet processed by accumulative roll bonding with the common roots and rapid annealing. J Mater Res Technol.

[20] Alizadeh M, Paydar MH, Terada D, Tsuji N (2012). Effect of SiC particles on the microstructure evolution and mechanical properties of aluminum during ARB process. Mater Sci Eng A.

[21] Jamaati R, Toroghinejad MR, Hoseini M, Szpunar JA (2011). Texture development in Al/Al_2_O_3_ MMCs produced by anodizing and ARB processes. Mater Sci Eng A.

[22] Hosseini M, Danesh Manesh H, Eizadjou M (2017). Development of high-strength, good-conductivity Cu/Ti bulk nano-layered composites by a combined roll-bonding process. J Alloys Compd.

[23] Roghani H, Borhani E, Jafarian HR, Yousefieh M, Naseri M, Ostovari Moghadam A (2023). On the impact of using alumina nanoparticles and initial aging on the microstructure and mechanical properties of the AA1050/AA2024 nanostructure composite, created by accumulative roll bonding (ARB) method. Wear..

[24] Mahdavian MM, Khatami-Hamedani H, Abedi HR (2017). Macrostructure evolution and mechanical properties of accumulative roll bonded Al/Cu/Sn multilayer composite. J Alloys Compd.

[25] Alizadeh M, Paydar MH, Sharifian Jazi F (2013). Structural evaluation and mechanical properties of nanostructured Al/B_4_C composite fabricated by ARB process. Compos Part B Eng.

[26] Paidar M, Ojo OO, Ezatpour HR, Heidarzadeh A (2019). Influence of multi-pass FSP on the microstructure, mechanical properties and tribological characterization of Al/B_4_C composite fabricated by accumulative roll bonding (ARB). Surf Coat Technol.

[27] Khdair AI, Fathy A (2020). Enhanced strength and ductility of Al-SiC nanocomposites synthesized by accumulative roll bonding. J Mater Res Technol.

[28] Ruppert M, Höppel HW, Göken M (2014). Influence of cross-rolling on the mechanical properties of an accumulative roll bonded aluminum alloy AA6014. Mater Sci Eng A.

[29] Lee SH, Saito Y, Sakai T, Utsunomiya H (2002). Microstructures and mechanical properties of 6061 aluminum alloy processed by accumulative roll-bonding. Mater Sci Eng A.

[30] Ghosh KS (2019). Calorimetric studies of 2024 Al–Cu–Mg and 2014 Al–Cu–Mg–Si alloys of various tempers. J Therm Anal Calorim.

[31] Chen K-C, Chao C-G (1995). Effect of δ alumina fibers on the aging characteristics of 2024-based metal-matrix composites. Metall. Mater. Trans. A..

[32] Tsuji N, Saito Y, Lee S-H, Minamino Y (2003). ARB (Accumulative Roll-Bonding) and other new techniques to produce bulk ultrafine grained materials. Adv Eng Mater.

[33] Tamimi S, Ketabchi M, Parvin N, Sanjari M, Lopes A (2014). Accumulative roll bonding of pure copper and IF steel. Int J Met.

[34] Jamaati R, Toroghinejad MR, Edris H (2013). Effect of stacking fault energy on nanostructure formation under accumulative roll bonding (ARB) process. Mater Sci Eng A.

[35] Cheng S, Zhao YH, Zhu YT, Ma E (2007). Optimizing the strength and ductility of fine structured 2024 Al alloy by nano-precipitation. Acta Mater..

[36] Lim SC, Ashby MF, Brunton JH (1987). Wear-rate transitions and their relationship to wear mechanisms. Acta Metall.

[37] Talachi AK, Eizadjou M, Manesh HD, Janghorban K (2011). Wear characteristics of severely deformed aluminum sheets by accumulative roll bonding (ARB) process. Mater Charact.

[38] Eizadjou M, Talachi AK, Manesh HD, Janghorban K (2011). Sliding wear behavior of severely deformed 6061 aluminum alloy by accumulative roll bonding (ARB) process. Mater Sci Forum.

[39] Darmiani E, Danaee I, Golozar MA, Toroghinejad MR, Ashrafi A, Ahmadi A (2013). Reciprocating wear resistance of Al–SiC nano-composite fabricated by accumulative roll bonding process. Mater Des.

[40] Khelge S, Kumar V, Shetty V, Kumaraswamy J (2022). Effect of reinforcement particles on the mechanical and wear properties of aluminium alloy composites: review. Mater Today Proc.

[41] Zhang Y, Jiang J, Wang Y, Xiao G, Liu Y, Huang M (2022). Recrystallization process of hot-extruded 6A02 aluminum alloy in solid and semi-solid temperature ranges. J Alloys Compd.

[42] Guarda C, Faria B, Silvestre N, Lopes JNC, Pugno NM (2022). Melted and recrystallized holey-graphene-reinforced aluminum composites: structure, elasticity and strength. Compos Struct.

